# Combining Fiber Enzymatic Pretreatments and Coupling Agents to Improve Physical and Mechanical Properties of Hemp Hurd/Wood/Polypropylene Composite

**DOI:** 10.3390/ma14216384

**Published:** 2021-10-25

**Authors:** Xiaoping Li, Mingli Qiang, Mingwei Yang, Jeffrey J. Morrell, Neng Zhang

**Affiliations:** 1 Yunnan Key Laboratory of Wood Adhesives and Glue Products, Southwest Forestry University, Kunming 650224, China; lxp810525@163.com (X.L.); qml2889@sohu.com (M.Q.); ymw1796233302@163.com (M.Y.); ZhangN329@163.com (N.Z.); 2 International Joint Research Center for Biomass Materials, Southwest Forestry University, Kunming 650224, China; 3 National Centre for Timber Durability and Design Life, University of the Sunshine Coast, Brisbane, QLD 4102, Australia

**Keywords:** pectinase, cellulase, polypropylene (PP), coupling agents, composites, wood fibers, hemp hurd fibers, cell wall ultrastructure

## Abstract

Natural fiber/plastic composites combine the low density and excellent mechanical properties of the natural fiber with the flexibility and moisture resistance of the plastic to create materials tailored to specific applications in theory. Wood/plastic composites (WPC) are the most common products, but many other fibers are being explored for this purpose. Among the more common is hemp hurd. Natural fibers are hydrophilic materials and plastics are hydrophobic, therefore one problem with all of these products is the limited ability of the fiber to interact with the plastic to create a true composite. Thus, compatibilizers are often added to enhance interactions, but fiber pretreatments may also help improve compatibility. The effects of pectinase or cellulase pretreatment of wood/hemp fiber mixtures in combination with coupling agents were evaluated in polypropylene panels. Pretreatments with pectinase or cellulase were associated with reduced thickness swell (TS_24h_) as well as increased modulus of rupture and modulus of elasticity. Incorporation of 5.0% silane or 2.5% silane/2.5% titanate as a coupling agent further improved pectinase-treated panel properties, but was associated with diminished properties in cellulase treated fibers. Combinations of enzymatic pretreatment and coupling agents enhanced fiber/plastic interactions and improved flexural properties, but the effects varied with the enzyme or coupling agent employed. The results illustrate the potential for enhancing fiber/plastic interactions to produce improved composites.

## 1. Introduction

Interest in natural fiber/plastic composites has increased as manufacturers search for products that can perform in exterior applications, such as decking, or provide exceptional strength properties [1,2,3,4,5,6,7]. Wood/plastic composites have received the most attention because they combine the low density and high tensile strength of wood with the moisture resistance of plastics, but a number of other cellulosic fibers have been explored for this purpose, including rice hull husks and hemp (fiber or hurd), in locations where wood is less abundant [8,9,10,11]. The one negative aspect of all these materials is the lack of substantial interactions between the hygroscopic fiber and hydrophobic plastic [12]. While compatibilizers such as silanes, titanate, and maleic anhydride can be added to enhance interactions, they add cost and still do not fully integrate the two materials. Identifying pretreatments for making fibers more compatible with the plastic might help improve properties [13,14,15,16].

Pretreatment with cell wall degrading enzymes such as pectinase or cellulase might improve the surface characteristics to render them more interactive with the plastic [17]. Pectin, cellulose, hemi-cellulose, and lignin are the main chemical compounds of plant cell walls. Pectin represents up to 35% of the primary cell wall and functions to bind the still developing cells together during secondary cell wall formation [18,19,20,21]. Its role in the mature cell wall remains unclear although there have been suggestions that its disruption can affect material properties [22,23]. Cellulose represents 40 to 50% of the secondary cell wall and its many hydroxyls play an important role in hygroscopicity and, therefore, the inability to interact with hydrophobic plastics. Enzymatic treatments to modify pectin or cellulose could change the distribution of chemical functional groups on the fiber surface resulting in improved physical and mechanical properties of the resulting composites [23].

There are few reports on the use of combinations of enzymatic pretreatment and coupling agents to improve wood plastic composite properties [8]. Enzyme pretreatment studies have mainly focused on analyzing the effects on surface chemical functional groups and residual chemical components, but less on the effects on fiber physical characteristics as they might affect plastic interactions [23]. Furthermore, another negative aspect of wood/plastic composites are their higher densities, which are twice those of ordinary wood materials (0.6–1.0 g/cm^3^) [24]. Reduced density of both wood/plastic composites and foamed wood/plastic composites have been studied and ranged from 0.62 g/cm^3^ to 0.96 g/cm^3^, with MORs ranging from 3.05 MPa to 32.08 MPa, mainly based on density of composites [25].

The objective of this study was to explore the potential for combining enzymatic pretreatments and coupling agents to improve the properties of wood/hemp hurd/polypropylene composite panels and create low-density, high strength wood/plastic composites.

## 2. Materials and Methods

### 2.1. Fiber Preparation

**Figure 1:**
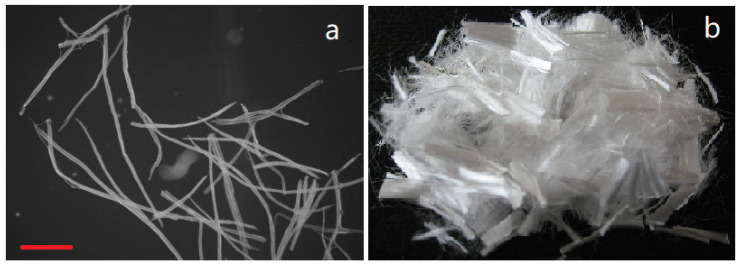
The Fibers used for composites in this experiment. (**a**) Hemp hurd/wood mixture fibers (Bar: 200 μm); (**b**) PP fibers.

Industrial hemp hurd obtained from the Yunnan Academy of Agricultural Sciences (Kunming, China), was cut into 5 to 10 mm wide by 10 to 20 mm long pieces and boiled at 100 °C for 1 h. The resulting material was macerated into fibers on a Yingte 2500-1 disc refiner (Yingte Naisen Precision Instruments, Dongguan, China). Wood fibers were obtained from a local medium density fiberboard (MDF) manufacturer. The resulting 2 to 3 mm long wood and hemp hurd fibers were both oven-dried at 104 °C and stored until needed. Previous studies have shown that mixtures of hemp hurd and wood can improve WPC properties [26]. The wood/hemp hurd fibers were thoroughly blended at a 70:30 ratio (oven-dry weight basis) for 60 min before being divided into 14 groups each containing 250 g of dry fiber (Figure 1a). Four groups were retained as non-modified controls that were only immersed in distilled water for one hour at 50 °C. These fibers were then dried at 104 °C prior to use. Five of the remaining ten 250 g batches were allocated to be pretreated with pectinase while the remainder were pretreated with cellulase.

### 2.2. Enzymatic Pretreatments

Pectinase (30,000 U/g) and cellulase (10,000 U/g) produced by *Aspergillus niger* fermentation were purchased from Shanghai Aladdin Biochemical Technology Co., Ltd. (Shanghai, China). The enzymes were diluted in separate solutions to a concentration of 0.05% (wt/wt) in distilled water (Pectinase about 15 U/mL, cellulase about 5 U/mL). Fifty liters of a given enzyme solution was added to each 250 g batch of dry fibers and thoroughly mixed. The resulting mixture was heated to 50 °C for 30 or 60 min with agitation to encourage mixing. At the end of the treatment period, the solution was decanted and the residual fibers were repeatedly washed with distilled water to remove residual enzyme. The fibers were oven-dried at 104 °C prior to use. Four 250 g batches of fibers were treated with pectinase for 30 min while an additional group was treated for 60 min. Four groups were immersed in the cellulase solution for 60 min while the remaining group was treated for 30 min (Table 1). The samples were all oven-dried and stored under dry conditions prior to use.

### 2.3. Panel Manufacturing

Polypropylene fibers (3 mm to 5 mm in length) (Figure 1b) with a melting temperature of 165–170 °C, tensile strength of 500 MPa, and modulus of elasticity of 3850 MPa were purchased from Shanxi Tongshenghua Engineering Technology Ltd. (Xi’an, China). Silane coupling agent KH570, titanate coupling agent KR-38S, and maleic anhydride were purchased from Shanghai National Medicine Group Chemical Reagent Co (Shanghai, China).

The pretreated wood/hemp material was mixed with the polypropylene at a 60:40 fiber/plastic ratio (wt/wt) along with the appropriate amount of coupling agent and dried at 80 °C (Table 1). The mixture was thoroughly blended and formed into 100 mm long by 10 mm wide mats that were pressed for 8 min at 180 °C to a target density of 0.75 g/cm^3^ and thickness of 2 mm. The samples were conditioned at 23 °C and 65% relative humidity for 48 h prior to testing. Fifteen samples were produced for each fiber treatment. Ten samples were used directly for flexural testing while the remaining five were cut into 50 by 10 by 2 mm thick samples for moisture sorption and thickness swelling measurements.

### 2.4. Water Uptake Properties

**Figure 2:**
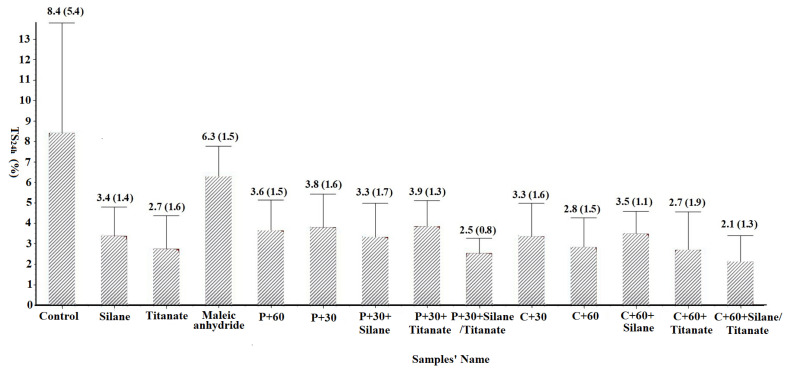
Effect of pectinase or cellulase pretreatment of a 70:30 hemp hurd/wood mixture on thickness swelling (TS) of composites manufactured using these materials and polypropylene. Values represent means of 10 replicates/test. Values in parentheses represent one standard deviation; C = cellulase, P = pectinase, and 30 or 60 represent exposure times in minutes.

**Figure 3:**
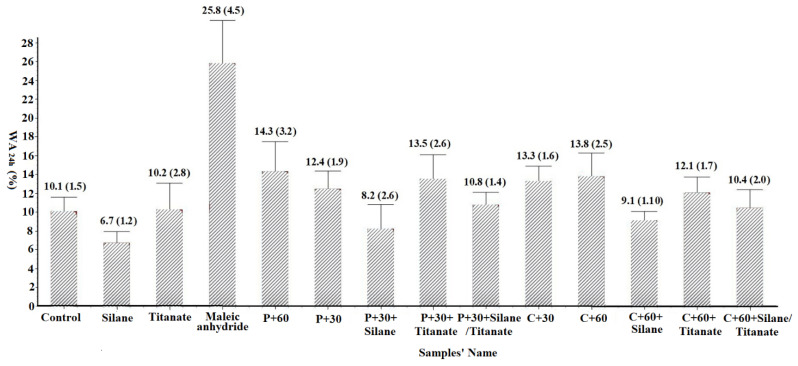
The water absorption for 24 h (WA_24h_) of composites (Values represent means of 10 replicates/test. Values in parentheses represent one standard deviation; the detail for samples’ name shown in Table 1).

Water uptake and thickness swell were determined by weighing each 50 by 10 mm sample then measuring its dimensions. The samples were immersed in water at room temperature (20 °C) for 24 h. The samples were weighed and dimensions were measured after 24 h of immersion. Differences between initial and final measurements were used to calculate water absorption and thickness swelling (Figure 2 and Figure 3), respectively [27].

### 2.5. Bending Properties

**Figure 4:**
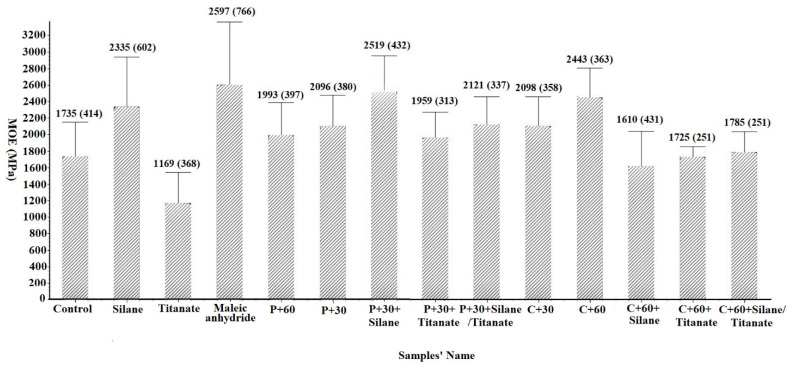
Elastic modulus of composites (MOE) (Values represent means of 10 replicates/test. Values in parentheses represent one standard deviation; the detail for samples’ name shown in Table 1).

**Figure 5:**
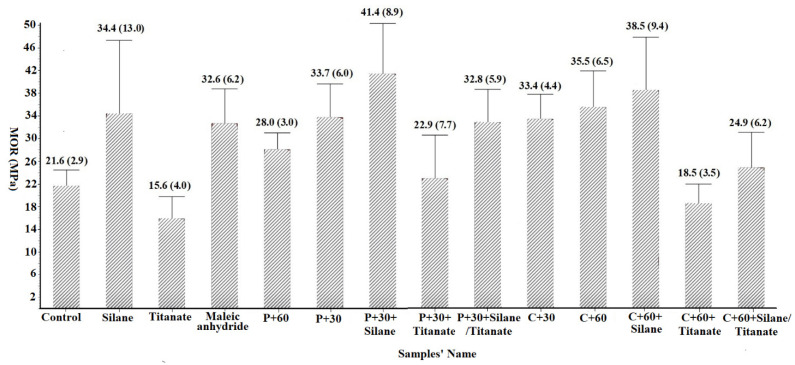
The Modulus of rupture (MOR) of composites (Values represent means of 10 replicates/test. Values in parentheses represent one standard deviation; the detail for samples’ name shown in Table 1).

The 100 by 10 by 2 mm thick samples were subjected to a third point bending test at a loading rate of 1 mm per minute to failure on a Universal Testing Machine, according to procedures described in ASTM Standard D790-02 [28]. The resulting load/deflection data were used to calculate modulus of elasticity (MOE) and modulus of rupture (MOR) (Figure 4 and Figure 5).

### 2.6. Fiber Characterization

**Figure 6:**
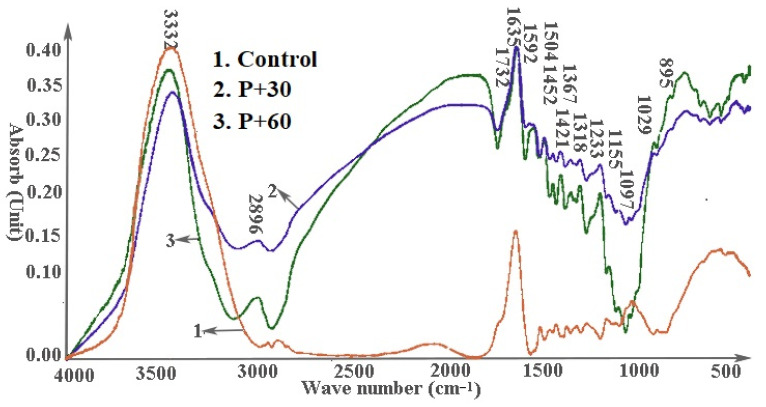
Effect of pectinase pretreatment on FTIR spectra of a 70:30 wood/hemp hurd fiber mixture.

**Figure 7:**
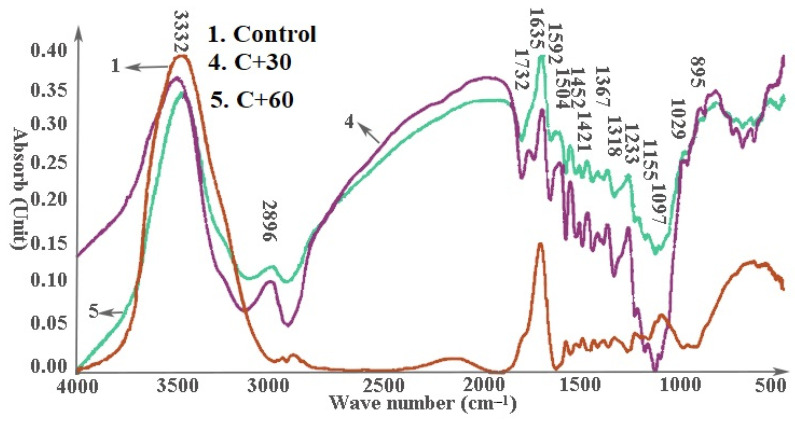
Effect of Cellulase pretreatment on FTIR spectra of a 70:30 wood/hemp hurd fiber mixture.

The effects of enzymatic treatment on fiber cell wall chemistry were studied using Fourier Transform Infrared Spectroscopy (FTIR). Sub-samples of the fiber mixture, treated with pectinase or cellulase for 30 or 60 min, along with the hot water treated control were ground to pass through a 200 mesh screen and the resulting fine powder was mixed with KBr, pressed into a pellet, and analyzed on a Nicolet i50 FTIR Analyzer (Thermo Scientific, Waltham, MA, USA). Samples were subjected to 64 scans and the resulting spectra were baseline corrected and then analyzed for differences in spectra between untreated and enzyme treated samples. Tentative peak identities were classified using previous literature (Table 2). Peak height ratios were used to compare results from different treatments (Figure 6 and Figure 7).

The ultrastructures of the hemp hurd cross-sections were observed by field emission scanning electron microscopy (FE-SEM: Nova NanoSEM 450 type, manufactured by FEI, USA). Control samples treated with distilled water at 50 °C for 60 min, samples treated with pectinase at 50 °C for 30 min, and samples treated with cellulase pectinase at 50 °C for 60 min were examined.

## 3. Results and Discussion

### 3.1. Effects of Fiber Pretreatment on Panel Moisture Behaviour

#### 3.1.1. Thickness Swelling for 24 h (TS_24h_)

TS_24h_ is an important index for evaluating wood/plastic composite stability. The largest thickness swell was 8.4% observed in the non-pretreated control panels with no coupling agent (Figure 2). The addition of a silane, titanate, or maleic anhydride coupling agent to non-pretreated fibers was associated with substantial decreases in thickness swell of 59.5%, 67.9%, and 25%, respectively, compared to the control.

Thickness swell of panels with 30 or 60 min pectinase or cellulase treated fibers decreased by 54.78%, 57.1%, 60.7%, and 66.7% respectively, compared with the non-pretreated control. Addition of silane or titanate also improved thickness swell (Figure 2).

Pectinase pretreatment for 30 min or cellulase pretreatment for 60 min also improved the mechanical properties of the composites and were selected to study potential synergistic effects between enzyme pretreatment and coupling agent (Figure 4 and Figure 5). Adding only one coupling agent did not markedly decrease thickness swell compared to the enzymatically pretreated fibers alone. However, TS_24h_ decreased by 75.0% for panels composed of cellulase pretreated fibers with 2.5% Silane/titanate. The greatest reductions in swelling were associated with both coupling agents and cellulase pretreatment, but the differences between pectinase and cellulase pretreatments were small. The effects of both enzymatic pretreatment and addition of coupling agents on properties are in line with the previous research [14,19,23]. The combination of these elements creates opportunities to further increase fiber/plastic compatibility to enhance panel properties. Coupling agents can improve compatibility between fiber surfaces and the plastic, improving water resistance, while enzymatic pretreatment can modify chemical groups on the fiber surface [23].

#### 3.1.2. Water Absorption for 24 h (WA_24h_)

Water absorption varied from 6.7% for the non-enzymatically treated panels with silane added to 25.8% for the non-pretreated panel with maleic anhydride (Figure 3). The addition of silane consistently reduced water uptake with reductions of 33.7%, 18.8%, and 9.9% for silane amended panels with non-pretreated, pectinase pretreated (30 min), or cellulase pretreated (60 min) fibers, respectively, compared to the similar panels without silane. This may be because the silane coupling agent and other organosilanes’ low surface energies and are good hydrophobic agents [33]. Although enzymatic pretreatment or addition of coupling agents should improve fiber/plastic interactions and potentially reduce moisture uptake [34], addition of titanate or maleic anhydride did not reduce moisture uptake (Figure 2). Low panel density (75 g/cm^3^) in these studies may have created micro-pathways that facilitated moisture intrusion, while the density of most WPC composites is higher than 1.2 g/cm^3^ [24].

### 3.2. Effects of Fiber Pretreatment on Panel Mechanical Properties

#### 3.2.1. Effect of Pretreatment on Elastic Modulus of Composites (MOE)

Addition of silane or maleic anhydride was associated with MOE increases for panels composed of non-modified fibers of 34.6% and 49.7%, respectively. These results are consistent with previous reports [14]. Enzymatic pretreatment was associated with increased MOE with the 30 min pretreatment producing higher values than the 60 min exposure for pectinase, while the 60 min pretreatment produced higher values than the 30 min exposure for cellulase. Cellulase pretreatment was associated with higher MOE values than pectinase. Silane addition to pectinase pretreated panels was associated with increased MOE’s of 7.88% and 20.18% compared with silane addition in non-treated fiber panels and pectinase pretreated panels (Figure 4). However, addition of titanate to untreated, pectinase pretreated, or cellulase pretreated panels was associated with decreased MOE (Figure 4). Addition of coupling agents to cellulase pretreated fibers was generally not associated with increased MOE compared to the pretreated fibers alone (Figure 4). The substantial improvements in MOE with the combination of enzymatic pretreatment and coupling agents suggests the potential for using combinations of pretreatment and coupling agents

#### 3.2.2. Effect of Fiber Pretreatment on MOR

MOR values ranged from 15.6 MPa for the non-pretreated controls with titanate coupling agent to 41.4 MPa for panels with pectinase pretreated fibers and silane coupling agent (Figure 5). Titanate addition was associated with lower MOR values for non-modified fibers and no noticeable difference when added to pectinase or cellulase pretreated fibers. Thus, addition of titanate produced no measurable improvement in flexural properties. Pectinase or cellulase pretreatment with or without addition of silane or maleic anhydride were generally associated with increased MORs compared to non-pretreatment controls. For example, addition of maleic anhydride was associated with a 50.9% MOR increase compared to the non-amended control. Silane addition was associated with higher MOR in both pectinase and cellulase pretreated panels which were 91.7% and 78.2% higher than the non-pretreated controls, respectively. Improved results with silane were consistent with the previous reports [23].

### 3.3. FTIR Analysis

Pretreatment with either pectinase or cellulase was associated with decreased O–H stretching of hydroxyl groups at 3332 cm^−1^ (Figure 6 and Figure 7), which may account for the decreased thickness swell (Figure 2). Peak heights tended to increase for all other bonds following enzyme treatment except for groups at 1097 cm^−1^ treated with the pectinase for 60 min or cellulase for 30 min. The results suggest that enzyme pretreatments exposed more functional groups on the fiber surfaces.

Enzyme pretreatment produced some differential effects depending on exposure time. Peaks at 3332 cm^−1^ (O–H stretching of bonded hydroxyl groups), 1029 cm^−1^ (C=O stretching vibration in cellulose, hemicelluloses and lignin), and 895 cm^−1^ (C–H deformation in cellulose) were all higher on samples treated with pectinase for 60 min compared to those treated for 30 min. Peaks at 1592 cm^−1^, 1504 cm^−1^, 1452 cm^−1^, 1421 cm^−1^, 1367 cm^−1^, 1318 cm^−1^, 233 cm^−1^, 1155 cm^−1^, and 1097 cm^−1^ corresponding to lignin and hemicelluloses were higher on samples treated for 30 min with pectinase than those treated for 60 min. The results suggest that pectinase exposed more cell wall material on the fiber surface, potentially improving interactions, especially with the more hydrophobic lignin component. Increased pretreatment time was also associated with increases in peaks at 1029 cm^−1^ and 895 cm^−1^ corresponding to C=O stretching of all three cell wall polymers and C–H deformation of cellulose, respectively [29,30,31,32]. These results support earlier results [35].

Peaks at 3332 cm^−1^ (O–H stretching of bonded hydroxyl groups) and 895 cm^−1^ (C–H deformation in cellulose) were higher on spectra from samples treated for 30 min with cellulase than those on samples treated for 60 min; but peaks at 1592 cm^−1^, 1504 cm^−1^, 1452 cm^−1^, 1421 cm^−1^, 1367 cm^−1^, 1318 cm^−1^, 1233 cm^−1^, 1155 cm^−1^, 1097 cm^−1^, and 1029 cm^−1^ (lignin, hemicelluloses) were higher on spectra from samples treated for 60 min. The results suggest that cellulase pretreatment exposed more cell wall polymers [35], which could enhance subsequent plastic interactions.

**Figure 8:**
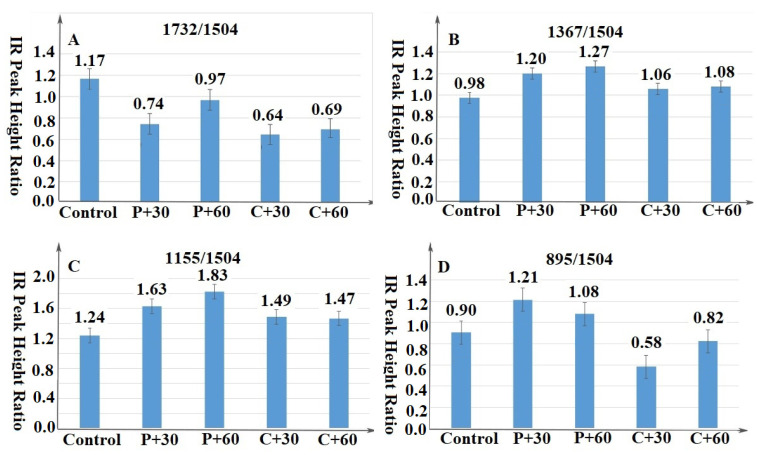
Effect of enzyme pretreatment on FT-IR spectra as shown by ratios between various carbohydrate fractions and lignin ((**A**) for 1732/1504; (**B)** for 1367/1504; (**C**) for 1155/1504 and (**D**) for 895/1504 respectively).

Delineating differences in peaks in a given spectrum can be difficult. One way to study differences is to choose relatively stable peaks across treatments and then compare peak height ratios. Peaks at 895, 1155, 1367, 1504, and 1732 cm^−1^ representing C–H deformation in cellulose, C–O–C vibration and C–H deformation in cellulose and hemicelluloses, C=C stretching in lignin, and C=O stretching in xylans, respectively, were chosen for comparison (Figure 8).

Ratios between the xylans peak at 1732 cm^−1^ and the lignin peak at 1504 cm^−1^ were reduced for all enzyme treatments with the greatest reduction in the two cellulase treatments suggesting that the enzymes selectively degraded hemicelluloses (Figure 8A). Ratios between the peak at 1367 cm^−1^ representing C–H deformation, and 1155 cm^−1^ reflecting C-H deformation in cellulose and hemicelluloses, and the peak at 1504 cm^−1^ increased with enzyme treatment (Figure 8B,C). While seemingly contradictory, these results are consistent with previous studies indicating reduced lignin content associated with these pretreatments [35], suggesting that the treatments affected lignin to a greater degree than carbohydrates. Finally, ratios between peaks at 895 cm^−1^ and 1504 cm^−1^ increased with pectinase treatment and decreased with cellulase treatment (Shown in Figure 8D). These trends are consistent with the ability of pectinase to degrade lignin, thereby reducing the ratio, while cellulase decreases the carbohydrate fraction but not the lignin, thereby increasing the ratio [7].

In general, pectinase and cellulase pretreatment were associated with losses of the corresponding polymers (lignin and cellulose, respectively), thus changing the composition and proportion of chemical functional groups on the fiber surface. This result is consistent with the previous results [35].

### 3.4. Effect of Fiber Pretreatment on Ultrastructure Hemp Hurd

**Figure 9:**
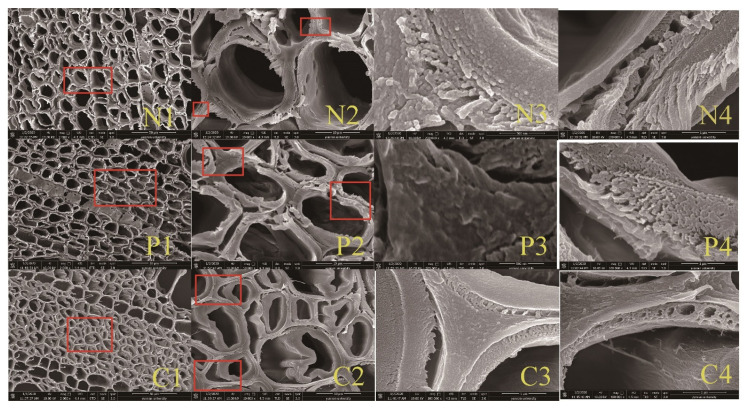
Scanning electron micrographs of cross sections cut from hemp hurd samples with no treatment (N1, N2, N3, and N4), exposed to pectinase for 30 (P1, P2, P3, and P4), or cellulase for 60 min (C1, C2, C3, and C4) and increasingly higher magnifications of the same fields.

Enzymatic treatments were intended to modify the cell wall surfaces to render them more exposed to potential polypropylene interactions (Figure 9). Pectinase should affect the more complex pectin and lignin polymers while the cellulase should expose lignin. Scanning electron microscopic examination of pectinase and cellulase treated hemp hurd suggested that the middle lamella of pectinase treated samples was separated from the adjacent cell walls, similar to previous research [36,37]. Cell separation would increase cell surface area, potentially exposing the more hydrophobic lignin to interact with the polypropylene. Examination of cellulase treated samples suggested some cell wall thinning in the secondary cell walls that could also increase lignin exposure along with separation between individual cells. Average cell wall thicknesses in control samples were 3.26 µm (from 1.60 µm to 5. 05 µm), while cell wall thickness in 30 min pectinase treated samples was 1.98 µm (from 1.06 µm to 3.19 µm) versus 2.21 µm (from 1.06 to 3.19 µm) for samples pretreated for 60 min with cellulase. The results suggested that enzyme pretreatment reduced cell wall thickness and may have contributed to improved polypropylene interactions.

## 4. Conclusions

Pectinase or cellulase pretreatments for different times produced inconsistent effects on both water resistance (water uptake and thickness swelling) and mechanical properties (MOE and MOR), but panels from these materials tended to perform better than panels composed of non-modified fibers. Enzymatic pretreatment can change the chemical groups on fibers’ surfaces and reduce the thickness of fibers’ cell walls, therefore a thinner cell wall is more effective for the penetration of plastics in biomass materials. There was a synergistic effect between pectinase pretreatment and silane coupling agent, but not between cellulase pretreatment and silane coupling agent. This may be because that the functional groups of carbohydrates on the surface of raw materials increase after pectinase pretreatment, and these functional groups are easier to form chemical bonds with silane coupling agents than lignin. The results suggest that combining pectinase pretreatment with silane addition may be used to manufacture one kind of low density fiber/plastic composites with super high properties.

## Figures and Tables

**Table 1:** Treatments applied to a 70/30 mixture of wood/hemp hurd fibers prior to panel manufacturing and ratios of the resulting fibers and coupling agents used to produce panels.

Pretreatment		Coupling Agent (%)			Samples’ Name
Enzyme	Time (min)	Silane	Titanate	Maleic Anhydride	
none	-	-	-	-	Control
	-	5.0	-	-	Silane
	-	-	5.0	-	Titanate
	-	-	-	5.0	Maleic anhydride
0.05% pectinase	60	-	-	-	P + 60
	30	-	-	-	P + 30
	30	5.0	-	-	P + 30 + Silane
	30	-	5.0	-	P + 30 + Titanate
	30	2.5	2.5	-	P + 30 + Silane/Titanate
0.05% cellulase	30	-	-	-	C + 30
	60	-	-	-	C + 60
	60	5.0	-	-	C + 60 + Silane
	60	-	5.0	-	C + 60 + Titanate
	60	2.5	2.5	-	C + 60 + Silane/Titanate

**Table 2:** Assignments of FTIR peaks to various cell wall polymer components.

Wave Number (cm^−1^)	Band Assignment	References
3332	O-H stretching of bonded hydroxyl groups	[29,30,31]
2896	Symmetric CH stretching in aromatic methoxyl groups and in methyl and methylene groups of side chains	[30,31]
1732	C=O stretching in xylans (unconjugated)	[30,31,32]
1635	H-O-H deformation vibration of absorbed water and C=O stretching in lignin	[30,32]
1592	C=C stretching of the aromatic ring (S)Aromatic skeletal vibrations + C=O stretching S ≥ G	[30,31,32]
1504	C=C stretching of the aromatic ring (G)Aromatic skeletal vibrations in lignin	[30,31,32]
1452	CH_2_ deformation vibrations in lignin and xylans	[30,31]
1421	C–H asymmetric deformation in –OCH_3_Aromatic skeletal vibrations combined with C-Hin plane deformation + C-H deformation in lignin and carbohydrates	[29,31,33]
1367	C-H deformation in cellulose and hemicelluloses	[29,30,31]
1318	C-H vibration in cellulose + C1-O vibration insyringyl derivatives	[30,31]
1233	Acetyl and carboxyl vibrations in xylans and C=O stretching vibrations in lignin	[30,31]
1155	C-O-C vibration in cellulose and hemicelluloses	[30,31]
1097	Aromatic C–H in-plane deformation (typical for S units), C=O stretch O-H association band in cellulose and hemicelluloses	[30,31]
1029	C=O stretching vibration in cellulose, hemicelluloses and lignin	[30,31]
895	C-H deformation in cellulose	[29,30,31]

## Data Availability

The data presented in this study are available from the listed authors.
